
JOURNAL INFORMATION
==============================
NLM Title Abbreviation: J Cardiothorac Surg
Journal ID: J Cardiothorac Surg
NLM Journal ID: 101265113

Journal of Cardiothoracic Surgery

EISSN: 1749-8090
Publisher: BMC

ARTICLE INFORMATION
==============================
PMCID: PMC5751388
DOI: 10.1186/s13019-017-0662-9
Article ID: 662
Article version: 1
Subjects: Meeting Abstracts

Abstracts of the 27th Congress of the World Society of Cardiovascular and Thoracic Surgeons (WSCTS)

Astana, Kazakhstan. 01-03 September 2017

Electronic publication date: 2017 Dec 15
Publication date: 2017
Volume: 12
Issue: Suppl 1
Issue sponsor: Publication of this supplement was funded by the Corporate Fund "Centr Serdca".
Electronic Location ID: 111
Copyright: © The Author(s). 2017
License: Open AccessThis article is distributed under the terms of the Creative Commons Attribution 4.0 International License (http://creativecommons.org/licenses/by/4.0/), which permits unrestricted use, distribution, and reproduction in any medium, provided you give appropriate credit to the original author(s) and the source, provide a link to the Creative Commons license, and indicate if changes were made. The Creative Commons Public Domain Dedication waiver (http://creativecommons.org/publicdomain/zero/1.0/) applies to the data made available in this article, unless otherwise stated.
License URL: https://creativecommons.org/licenses/by/4.0/

Conference name: 27th Annual Congress of the World Society of Cardiovascular and Thoracic Surgeons (WSCTS)
Conference Acronym: WSCTS 2017
Conference location: Astana, Kazakhstan
Conference date: 01-03 September 2017
issue-copyright-statement: © The Author(s) 2017

==============================
Publisher’s Note

Springer Nature remains neutral with regard to jurisdictional claims in published maps and institutional affiliations.

